# Association of Jail Decarceration and Anticontagion Policies With COVID-19 Case Growth Rates in US Counties

**DOI:** 10.1001/jamanetworkopen.2021.23405

**Published:** 2021-09-02

**Authors:** Eric Reinhart, Daniel L. Chen

**Affiliations:** 1 Data and Evidence for Justice Reform, The World Bank, Washington, DC; 2 Department of Anthropology, Harvard University, Cambridge, Massachusetts; 3 Department of Psychiatry and Behavioral Sciences, Northwestern University, Chicago, Illinois; 4 Centre National de la Recherche Scientifique, Paris, France; 5 Toulouse School of Economics, Toulouse, France; 6 Institute for Advanced Study in Toulouse, Toulouse, France

## Abstract

**Importance:**

Mass incarceration is known to foster infectious disease outbreaks, amplification of infectious diseases in surrounding communities, and exacerbation of health disparities in disproportionately policed communities. To date, however, policy interventions intended to achieve epidemic mitigation in US communities have neglected to account for decarceration as a possible means of protecting public health and safety.

**Objective:**

To evaluate the association of jail decarceration and government anticontagion policies with reductions in the spread of SARS-CoV-2.

**Design, Setting, and Participants:**

This cohort study used county-level data from January to November 2020 to analyze COVID-19 cases, jail populations, and anticontagion policies in a panel regression model to estimate the association of jail decarceration and anticontagion policies with COVID-19 growth rates. A total of 1605 counties with data available on both jail population and COVID-19 cases were included in the analysis. This sample represents approximately 51% of US counties, 72% of the US population, and 60% of the US jail population.

**Exposures:**

Changes to jail populations and implementation of 10 anticontagion policies: nursing home visitation bans, school closures, mask mandates, prison visitation bans, stay-at-home orders, and closure of nonessential businesses, gyms, bars, movie theaters, and restaurants.

**Main Outcomes and Measures:**

Daily COVID-19 case growth rates.

**Results:**

In the 1605 counties included in this study, the mean (SD) prison population was 283.38 (657.78) individuals, and the mean (SD) population was 315.24 (2151.01) persons per square mile. An estimated 80% reduction in US jail populations, achievable through noncarceral management of nonviolent alleged offenses and in line with average international incarceration rates, would have been associated with a 2.0% (95% CI, 0.8%-3.1%) reduction in daily COVID-19 case growth rates. Jail decarceration was associated with 8 times larger reductions in COVID-19 growth rates in counties with above-median population density (4.6%; 95% CI, 2.2%- 7.1%) relative to those below this median (0.5%; 95% CI, 0.1%-0.9%). Nursing home visitation bans were associated with a 7.3% (95% CI, 5.8%-8.9%) reduction in COVID-19 case growth rates, followed by school closures (4.3%; 95% CI, 2.0%-6.6%), mask mandates (2.5%; 95% CI, 1.7%-3.3%), prison visitation bans (1.2%; 95% CI, 0.2%-2.2%), and stay-at-home orders (0.8%; 95% CI, 0.1%-1.6%).

**Conclusions and Relevance:**

Although many studies have documented that high incarceration rates are associated with communitywide health harms, this study is, to date, the first to show that decarceration is associated with population-level public health benefits. Its findings suggest that, among other anticontagion interventions, large-scale decarceration and changes to pretrial detention policies are likely to be important for improving US public health, biosecurity, and pandemic preparedness.

## Key Points

**Question:**

Were jail decarceration and government implementation of anticontagion policies associated with the spread of SARS-CoV-2 in US counties?

**Findings:**

In this cohort study of 1605 counties in panel regression models, an estimated 80% reduction in US jail populations would have been associated with a 2% reduction in daily COVID-19 case growth rates, with considerably greater COVID-19 reductions in counties with above-median population density and above-median proportion of Black residents. In analyses of anticontagion policies, nursing home visitation bans were associated with a 7.3% reduction in COVID-19 growth rates, followed by school closures (4.3%), mask mandates (2.5%), and prison visitation bans (1.2%).

**Meaning:**

The findings of this study suggest that anticontagion policies, including jail decarceration to minimize carceral outbreaks and their spillover to surrounding communities, appear to be necessary for epidemic control, public health, and mitigation of racial health disparities.

## Teaser

This case-control study examines the outcomes associated with jail decarceration during the COVID-19 pandemic.

## Introduction

Anticontagion policies have been unevenly implemented across jurisdictions in the US during the COVID-19 pandemic, presenting an opportunity for a natural experiment with which to evaluate their outcomes.^1^ Although several studies have provided provisional analyses, the consequences associated with anticontagion policies remain largely unknown.^2,3,4,5,6,7,8^ In some cases, policy decisions have drawn on international studies that may not accurately reflect distinctive biosocial dynamics shaping epidemiologic characteristics in the US.^9,10,11,12,13,14,15^ Furthermore, some proposed anticontagion measures, such as reducing jail populations and the associated cycling of detainees through crowded carceral facilities that appear to pose high risks for SARS-CoV-2 spread in communities,^16,17,18,19^ are currently understood only through computer simulations and require further evaluation with empirical evidence.^20^

Although widely neglected by policy makers both domestically and internationally, jails and prisons are sites of major epidemiologic importance for public health, pandemic preparedness, and biosecurity.^16,17,18,19,20^ As of September 1, 2020, despite inadequate testing and reporting in many jails and prisons, these facilities represented 90 of the top 100 COVID-19 clusters in the US.^21^ Incarcerated individuals have faced 5.5 times higher risk of contracting COVID-19 than those in the general US population and, after adjusting for age, sex, and race/ethnicity, 3 times the COVID-19 mortality rate.^22^ Because SARS-CoV-2 testing, health care infrastructure, data collection and transparency, and auditing and supervisory structures have been inadequate in US jails and prisons, the true risks to detainees may be considerably higher than documented.^16,18,23^ These risks to incarcerated persons have motivated calls for increased compassionate releases and decarceration measures consisting of both large-scale releases and front-end diversion away from initial incarceration.^16,24^ To this point, however, policy makers and criminal punishment administrators have neglected to adequately address the pandemic crisis in carceral facilities.^16,25^

COVID-19 outbreaks in jails, prisons, and immigrant detention facilities do not only pose risks to incarcerated people, they also appear to spread to surrounding communities.^16,17,18,19,20^ This carries particularly pronounced consequences for Black and Latinx communities that are subjected to disproportionately high rates of arrest and incarceration, which may partially explain the disproportionate burden of COVID-19 that has been borne by racialized groups in the US.^18^

Carceral-community epidemiologic relationships, that is, connections between carceral conditions and disease spread in broader communities, have long been observed worldwide in relation to, for example, HIV, tuberculosis, influenza, and viral hepatitis.^16,17,18,19,26,27,28,29,30,31,32^ To date, however, only 2 modeling studies,^20,33^ 2 peer-reviewed studies of empirical evidence limited to Illinois,^17,18^ and 1 non–peer-reviewed empirical analysis^34^ have specifically examined the association between carceral institutions and community spread of SARS-CoV-2. Although there is a growing body of empirical literature on the consequences of mass incarceration on community health,^17,18,31,35,36^ to our knowledge, no study has yet evaluated the effects of decarceration on population-level community health outcomes, either in relation to COVID-19 or otherwise.

Given the flow of approximately 200 000 detainees through US jail facilities every week and the daily commutes of more than 220 000 full-time jail staff,^37^ jails in particular—compared with prisons, which house those convicted of charges and serving sentences longer than 1 year and which feature relatively less dynamic populations—have high potential to function as infectious disease reservoirs and epidemiologic pumps that fuel COVID-19 incidence in surrounding communities. The US jail population, 75% of which is composed of pretrial detainees and 25% of individuals sentenced to less than 1 year for minor offenses,^37^is in constant biosocial interrelation with surrounding communities.^16,17,18,19,35,36^

It is thus especially concerning that a study of a large urban jail demonstrated the highest known institutional SARS-CoV-2 basic reproduction ratio (8.44) observed in any context to date.^38^ Such rapid viral spread in overcrowded US jails,^39^ constant flow of detained people and staff, inadequate testing,^16^ and the high rate of detainee turnover (55% of the US jail population turns over each week^33^) suggest that rapid spread of SARS-CoV-2 among those detained, often for only a matter of days, is likely to be disseminating into their home communities following release.^16,17,18,19,40^ In this context, this study presents an analysis of the association between jail decarceration and anticontagion policies, included both as potential confounders in our analysis of decarceration and as interventions of interest in their own right, with daily growth rates in COVID-19 cases in US counties.

## Methods

### Data Collection

We examined the epidemiologic association between anticontagion measures and COVID-19 at the county level using data on jail populations, anticontagion policies, and COVID-19 cases. Jail population data (January 1 to November 15, 2020) were obtained from the Vera Institute of Justice and represent 1614 counties in the US.^41^ Our sample included 1605 counties with data available on both jail population and COVID-19 cases, resulting in 51% of US counties, 72% of the US population, and 60% of the US jail population. COVID-19 case data were obtained from *The New York Times*.^42^ This study used only deidentified public sources and was deemed exempt from institutional review board approval under guidelines at Harvard University. This study followed the relevant sections of the Strengthening the Reporting of Observational Studies in Epidemiology (STROBE) reporting guideline.

We merged data on policy interventions from the COVID-19 US State Policy Database^43^ supplemented with additional county-level data aggregated by the Becker Friedman Institute,^44^ Stanford Intervention Data,^45^ and Keystone Strategy.^46^ We included the following policy interventions as covariates in our analysis: nursing home visitation bans, school closures, mask mandates, prison visitation bans, stay-at-home orders, and closure of nonessential businesses, gyms, bars, movie theaters, and restaurants. Interventions made at the state level were assigned to each county in that state. We included county-specific, non–COVID-19 US state policy–reported interventions for 1144 counties—all instances in which such data were available. eAppendix 1 in the Supplement provides more details.

To allow further interpretation, as an addition to our main analysis, we used average weekly detainee turnover rates reported by the Bureau of Justice Statistics, matched to jail size, to infer daily jail cycling (ie, the number of individuals arrested and cycled through jails with typical stays of only days to weeks before release) from data on daily jail populations.^37^ Demographic data, including race/ethnicity variables Black alone and Hispanic (inclusive of all categories) for testing heterogeneities and describing our study sample, were drawn from the 2018 American Community Survey and 2010 US Census.^47^

### Statistical Analysis

We used reduced-form econometric techniques commonly used to measure the association between policies and economic growth rates. Economic output, like SARS-CoV-2 infections, generally increases exponentially with a variable rate that can be affected by policies and other conditions. Our technique, which used panel regression models that have also been used by other researchers to analyze anticontagion policies associated with COVID-19 growth,^9^ measures the association of changes in policy and jail populations with COVID-19 case growth rates without requiring prior information about fundamental epidemiologic parameters or mechanisms.

To construct the dependent variable, we transformed county-specific time-series data on COVID-19 cases into first differences of their natural logarithm (specifically, natural logarithm transformation on cases plus 1), which is the per-day growth rate of cases. We used panel regression models to estimate how the daily growth rate of cases changed over time within a county with respect to changes in jail populations and policy interventions in that county. Our econometric approach—a standard method in economic literature when using longitudinal data—controls for differences in the baseline growth rate of COVID-19 cases across counties that may be affected by time-invariant characteristics (ie, county-fixed effects), such as demographics, socioeconomic status, culture, and health systems.^9^ Standard errors were clustered at the state level. All hypothesis tests were 2-sided.

In addition to our main analysis, we analyzed 4 demographic subsets preselected based on questions we had at the outset of the study on differential magnitudes of the association between incarceration and COVID-19: above and below median proportion of population that identifies as Black, above and below median income, above and below population density, and the 50 most populous US counties. The first 2 subsets were selected based on the fact that US criminal legal system is known to disproportionately affect poor and racialized populations, especially Black communities, and to treat these populations differently across a range of criminal legal procedures that may affect the likelihood of SARS-CoV-2 exposure and spread.^48,49,50,51^ The last 2 subsets were selected because population density is known to be associated with more rapid infectious disease transmission and epidemiologic dynamics in US population centers, which carry significant import for US economic output and political power and may be of particular interest to policy makers who hold power to implement county- or city-specific public health interventions.

We examined cross-sectional models, changed the lag structure (ie, assumed a different temporal delay from jail population or policy change to changes in daily COVID-19 growth, which will be subject to both SARS-CoV-2 incubation periods and possibly delayed adherence to policy or behavioral changes following policy implementation), and display the data in visual form as raw data without controls (ie, without county-fixed effects) as well as in an analysis of a polynomial relationship between jail population and COVID-19 growth. This presentation of the raw data allows evaluation of whether collinearity or other unusual distributions of the raw data (eg, outliers) are affecting our results.

To explore more closely the polynomial relationship that appears in the robustness check, we analyzed 2 more subsets of the data based on epidemic intensity and different periods in the epidemic. To address concerns of omitted variables that affect jail populations within counties over time, we also assessed the association between mass releases from jails (using data from UCLA COVID-19 Behind Bars Data Project)^52^ with subsequent jail populations and COVID-19 growth rates. Mass release events constitute a sudden change to the ordinary pattern on the level of jail cycling, which allowed us to better assess potential associations between jail cycling and COVID-19 growth rates by addressing possible concerns for omitted variable bias. Additional robustness checks are described in eAppendix 1 in the Supplement. Significance thresholds are reported for 0.10, 0.05, and 0.001; all significance tests were 2-sided.

Our analyses were performed using the statistical modules available in R, version 4.0.3 (2020-10-10; R Project for Statistical Computing) and Stata, version 16 (StataCorp LLC).

## Results

**Figure 1.**
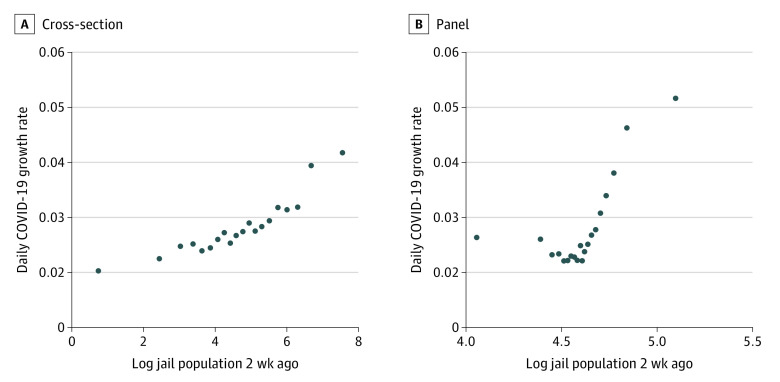
Association Between COVID-19 Daily Growth Rate and Log Daily Jail Population Controlling for County-Fixed Effects Both scatterplots show associations between COVID-19 growth rate and jail population. A, Controlled for county-fixed effects. B, Not controlled for county-fixed effects.

In the 1605 counties included in this study, the mean (SD) prison population was 283.38 (657.78) individuals, and the mean (SD) population was 315.24 (2151.01) persons per square mile. Table 1 provides details on analyzed variables, including jail population, inferred jail cycling, anticontagion policies, and key demographic considerations for counties in our sample along with summary statistics. The bivariate association of 1.9% in cross-section and 0.3% in panel analysis between COVID-19 daily growth rate and log daily jail population are presented in Figure 1 in 2 binned scatterplots—a visualization that is less restrictive than assuming a functional form. This presentation helps to visually address concerns of outliers and statistical significance by showing the patterns nonparametrically. Figure 1 plots the bivariate association with the log of the jail population with COVID-19 growth without controls (ie, the cross-sectional association between jail population and COVID-19 growth) and in a panel analysis with county-fixed effects. These scatterplots show that in the study sample, the daily jail population was significantly and positively associated with COVID-19 daily growth rates across counties. When controlled for fixed characteristics of counties, jail population remained positively and significantly associated with COVID-19 daily growth rate. Outliers did not appear to affect the pattern, and patterns for jail populations appear without controls for any policy variables. A polynomial relationship—more specifically, a quadratic relationship—seems to appear in the raw data, consistent with previous analyses indicating that reductions in jail population were associated with a decrease in transmission rates among detainees, which would in turn reduce the associated risk of spread of jail-acquired SARS-CoV-2 infections to surrounding communities.^53^

**Table 1.** Summary Statistics

Variable^a^	Observations by county by day, mean (SD) (N = 319 084)
Growth rate of COVID-19 cases	1.03 (0.12)
Jail incarcerated population, individuals	283.38 (657.78)
Estimated daily jail cycling, individuals	0.97 (19.79)
Nursing home visitation ban policy in effect	0.73 (0.44)
School closure policy in effect	0.97 (0.18)
Mask policy in effect	0.40 (0.49)
Prison visitation ban policy in effect	0.92 (0.27)
Stay-at-home policy in effect	0.19 (0.39)
Other nonessential business closure policy in effect	0.31 (0.46)
Gym closure policy in effect	0.19 (0.39)
Bar closure policy in effect	0.54 (0.50)
Movie theater closure policy in effect	0.38 (0.49)
Restaurant closure policy in effect	0.23 (0.42)
Percentage of population that identifies as Black	0.11 (0.14)
Population density, persons per square mile	315.24 (2151.01)

^a^ Policy variables have only binary values that are reflected in their mean. Value of 1 indicates policy was in effect on that particular date.

In multivariate panel regressions presented in Table 2, there persists both significant positive association and significant quadratic relationship between daily jail population and COVID-19 growth rates. During our sampling period, we estimate that reducing jail population by 80% would have been associated with a 2.0% (95% CI, 0.8%-3.1%) reduction in daily COVID-19 growth rates (calculated from the quadratic specification in Table 2). Of all anticontagion measures analyzed, prohibition of nursing home visitation had the largest negative association with COVID-19 growth, corresponding to a 7.3% (95% CI, 5.8%-8.9%) reduction in daily growth rates, followed by closure of schools (4.3%; 95% CI, 2.0%-6.6%), state-wide mandatory mask-wearing policy in public places (2.5%; 95% CI, 1.7%-3.3%), prison visitation bans (1.2%; 95% CI, 0.2%-2.2%), and stay-at-home policies (0.8%; 95% CI, 0.1%-1.6%). The other policy interventions analyzed were not significantly associated with COVID-19 growth rates, with the notable exceptions of restaurant closure policies, which were associated with an increase of COVID-19 daily growth rates by 2.2% (95% CI, 1.1%-3.3%).

**Table 2.** Estimated Associations Between COVID-19 Daily Growth Rate and Log Daily Jail Population and Anticontagion Policies in Multivariate Regression Analysis With County Fixed Effects

Independent variable	Coefficients for growth rate of COVID-19 cases	
	Quadratic (95% CI)	Linear (95% CI)
Log daily jail population 2 wk ago	−0.020 (−0.031 to −0.008)^a^	0.008 (0.003 to 0.012)^b^
Log daily jail population squared 2 wk ago	0.004 (0.002 to 0.006)^b^	NA^c^
Nursing home visitation ban policy in effect 2 wk ago	−0.073 (−0.088 to −0.058)^b^	−0.074 (−0.089 to −0.058)^b^
Schools closure policy in effect 2 wk ago	−0.043 (−0.066 to −0.020)^b^	−0.046 (−0.069 to −0.022)^b^
Mandatory mask policy (state-wide) in effect 2 wk ago	−0.025 (−0.033 to −0.017)^b^	−0.025 (−0.033 to −0.017)^b^
Prison visitation ban policy in effect 2 wk ago	−0.012 (−0.022 to −0.003)^d^	−0.013 (−0.023 to −0.003)^a^
Stay-at-home policy in effect 2 wk ago	−0.008 (−0.016 to 0.001)^e^	−0.008 (−0.017 to 0.001)^e^
Other nonessential businesses closure policy in effect 2 wk ago	−0.005 (−0.018 to 0.007)	−0.006 (−0.018 to 0.007)
Gyms closure policy in effect 2 wk ago	−0.005 (−0.013 to 0.003)	−0.006 (−0.014 to 0.002)
Bars closure policy in effect 2 wk ago	−0.003 (−0.011 to 0.005)	−0.003 (−0.010 to 0.005)
Movie theaters closure policy in effect 2 wk ago	−0.001 (−0.009 to 0.007)	−0.002 (−0.010 to 0.007)
Restaurants closure policy in effect 2 wk ago	0.022 (0.011 to 0.033)^b^	0.023 (0.012 to 0.035)^b^

Abbreviation: NA, not applicable.

^a^ *P* < .01.

^b^ *P* < .001.

^c^ Linear model will not have a coefficient for the quadratic term.

^d^ *P* < .05.

^e^ *P* <  .10.

Table 2 includes data that, when controlling for anticontagion policies, indicate that the daily jail population is associated with COVID-19 daily growth rates both quadratically and linearly. The association of jail population and COVID-19 daily growth rates appeared to grow quadratically, and eTable 1 in the Supplement shows that there is also a significant positive association with the cubic term.

Table 3 presents the associations of jail population reductions as a result of mass release events with COVID-19 daily growth rates and log daily jail population.^43^ When controlling for anticontagion policies, mass release events were associated with a 3.1% (95% CI, 1.9% to 4.3%) decrease in COVID-19 growth rates 2 weeks later and a 5.3% (95% CI, −3.5% to 14.1%) decrease in daily jail population. These patterns suggest that decreases in jail population following concerted decarceration efforts are associated with decreases in county-level COVID-19 daily growth rates.

**Table 3.** Estimated Associations Between COVID-19 Daily Growth Rate and Log Daily Jail Population Regressed on Jail Mass Release Events and Anticontagion Policies in Multivariate Analysis With County Fixed Effects

Independent variable	Coefficients for growth rate of COVID-19 cases (95% CI)	Log daily jail population 2 wk ago (95% CI)
Jail mass release event 2 wk ago	−0.031 (−0.043 to −0.019)^a^	−0.053 (−0.141 to 0.035)
Nursing home visitation ban in effect 2 wk ago	−0.030 (−0.066 to 0.006)^b^	0.084 (0.015 to 0.153)^c^
School closure policy in effect 2 wk ago	−0.066 (−0.093 to −0.040)^a^	−0.133 (−0.171 to −0.094)^a^
Mandatory mask policy (state-wide) in effect 2 wk ago	−0.020 (−0.031 to −0.008)^d^	−0.038 (−0.117 to 0.041)
Prison visitation ban policy in effect 2 wk ago	−0.011 (−0.030 to 0.009)	−0.097 (−0.138 to −0.056)^a^
Stay-at-home policy in effect 2 wk ago	−0.006 (−0.016 to 0.005)	−0.036 (−0.079 to 0.006)^b^
Other nonessential businesses closure policy in effect 2 wk ago	−0.016 (−0.023 to −0.008)^a^	−0.061 (−0.133 to 0.010)^b^
Gym closure policy in effect 2 wk ago	−0.004 (−0.013 to 0.005)	−0.074 (−0.147 to −0.000)^c^
Bar closure policy in effect 2 wk ago	0.007 (−0.002 to 0.017)	0.021 (−0.039 to 0.082)
Movie theater closure policy in effect 2 wk ago	−0.0095 (−0.0163 to −0.0026)^d^	−0.0623 (−0.1238 to −0.0009)^c^
Restaurant closure policy in effect 2 wk ago	0.010 (−0.005 to 0.024)	0.102 (0.039 to 0.164)^d^

^a^ *P* < .001.

^b^ *P* < .10.

^c^ *P* < .05.

^d^ *P* < .01.

**Figure 2.**
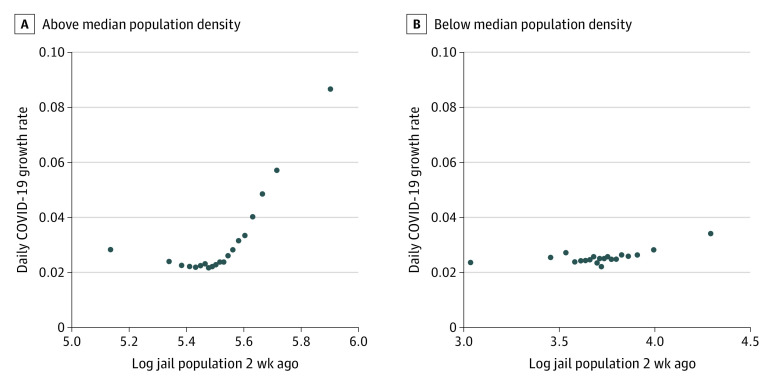
Between Jail Population and Growth in COVID-19 Growth in COVID-19 was larger in counties with above median population density (A) compared with counties with below median population density (B). The bivariate association between log daily jail population 2 weeks ago and COVID-19 growth with county-fixed effects for the 2 samples are plotted.

Figure 2 and eFigure 1 in the Supplement present binned scatterplots for 4 subsamples of the data: above and below the median proportion of Black residents, above and below the median income, above and below the median population density, and the 50 most populous US counties. We found that the linear relationship between daily jail population and COVID-19 daily growth rates was larger in each of these subsamples relative to our main sample (Figure 1). In counties with above-median population density, the association between jail population and COVID-19 growth rates (4.6%; 95% CI, 2.2% to 7.1%) was more than 8 times larger than in counties with below-median population density (0.5%; 95% CI, 0.1% to 0.9%). This rate was 3.2 times as large in counties with above-median household income (3.2%; 95% CI, 1.6% to 4.9%) relative to those below this median (1.0%; 95% CI, 0.3% to 1.6%) and 1.5 times larger in counties with above-median proportion of Black residents (2.4%; 95% CI, 1.1% to 3.6%) relative to those below this median (1.6%; 95% CI, 0.6% to 2.6%). For the 50 most populous counties, the association between jail population and COVID-19 growth rates (2.8%; 95% CI, −3.2% to 8.8%) was 1.5 times larger than in all other counties (1.9%; 95% CI, 0.1% to 2.8%).

eTable 2 in the Supplement repeats the regressions from Table 2, using inferred daily jail cycling instead of daily jail population. The estimated coefficient for jail cycling is twice as large compared with the analysis of jail population in Table 2, suggesting that the anticontagion implications of reducing jail cycling (ie, the throughflow of detainees) are substantially larger than those associated with reducing overnight jail populations alone. Additional robustness checks are described and their results reported in eTables 3 to 7 and eFigures 2 to 4 in the Supplement.

## Discussion

The results of this study suggest that jail decarceration and several anticontagion policies—nursing home and prison visitation prohibitions, school closures, and mask mandates—were associated with the prevention of a large number of COVID-19 cases. These policies may have been even more successful if implemented more widely.

Pandemic mitigation strategies should be reevaluated in light of increasingly available evidence on the relative associated harms and benefits of various anticontagion policies.^54,55^ Although business, nursing home, prison visitation, and school restrictions, for example, are associated with the reduction of COVID-19 growth rates, each may also entail negative tradeoffs, ranging from economic hardship and indirect morbidity and mortality to substantial mental health consequences and social harms to communities.^56,57,58,59,60,61,62,63,64,65^ Mask mandates and reducing jail populations also appear to be useful interventions and, by contrast, do not appear to entail such negative tradeoffs.^16^ In fact, existing evidence suggests that reducing reliance on jails for the management of minor alleged offenses would likely yield substantial benefits for adult and child mental health,^66,67^ short- and long-term economic opportunities,^68^ various social and public health benefits,^69,70,71^ and public safety.^19,72,73,74^ In addition, more than 80% reductions in jail populations, which was the preselected target we used that would bring the US closer to the 85% reduction in its incarceration rate required to match averages among peer nations, may be achieved simply by managing nonviolent alleged offenses through alternatives to incarceration.^75,76^ (It is also important to note in any discussion of the management of violent and nonviolent offenses in the US legal system that critical reevaluation of excessively long sentences for those convicted of violent offenses is an important—and neglected because politically unpopular—subject in need of evidence-based policy redress.^25^)

Our findings with respect to jail decarceration add to a growing body of literature on carceral-community epidemiology that documents the various ways in which the health and welfare of incarcerated people are intertwined with community health.^16,17,18,19,20,26,27,28,29,30,31,32,33,34,35,36,37,70,71,77,78^ Carceral outbreaks during the COVID-19 pandemic underline these studies’ observations that it is in the immediate and long-term interest of US public health and safety to confront high rates of incarceration and poor carceral conditions. Our findings thus support existing consensus among public health experts that large-scale decarceration is needed not only to mitigate the spread of SARS-CoV-2 but also, in the longer-term, to assist in remedying US racial health inequities and to improve national public health, pandemic preparedness, and international biosecurity.^16,18,24,71,78,79,80^

### Limitations

This study has limitations. Although panel regression models are helpful in addressing concerns of omitted variables, because this was a panel regression model using econometric techniques, we cannot determine causality. Our analysis necessarily relied on the assumption that jail admissions and releases are unrelated to omitted factors that may be associated with changes in COVID-19 cases within counties. However, other factors behind COVID-19 cases growth rates are likely associated with the county-level fixed effects and anticontagion policies for which we have controlled, and we found that these controls did not account for the association we observed between jail population and growth rates of COVID-19 cases.

A further limitation follows from our lack of access to data on jail staff. The more than 220 000 staff who move in and out of jails on a daily basis are likely to contribute to jail-community spread of airborne pathogens, such as SARS-CoV-2. Had we been able to account for this movement, we expect that association of jails with community COVID-19 case rates would be higher than captured by our present analysis.

Although jail population turnover (ie, releases and admissions) is relevant to the spillover of carceral outbreaks into broader communities, we are limited by access to data on daily jail populations without means of directly identifying detainee turnover between days. To enable interpretation nearer to this epidemiologic dynamic, we inferred daily jail cycling figures by imputing jail turnover statistics, matched to jail size, reported by the Bureau of Justice Statistics.^37^ During the COVID-19 pandemic, jail turnover rates have increased in some jurisdictions and decreased in others, leaving it unclear whether reliance on prepandemic turnover statistics may lead to bias in one direction or the other. We thus excluded inferred jail cycling from our primary specifications.

In terms of generalizability, our analysis included 51% of US counties, 72% of the US population, and 60% of the US jail population. This considerable sample size and its geographic breadth make the activities we observed broadly relevant even if they are not representative of all US jurisdictions. In addition, the associations we observed did not seem to be affected by outliers, which alleviates concerns that sampling factors biased our results, and were persistent in analyses of cross-sectional data, panel regressions, raw data, and multiple regressions with controls.

We were also limited by the nonuniversal coverage of data on county-level policy interventions. We describe this limitation in more detail in eAppendix 2 in the Supplement.

## Conclusions

The consequences of government policies to mitigate spread of infectious diseases during epidemic outbreaks have been highly contested throughout the COVID-19 pandemic. In this context, the absence of strong federal public health policies in the US has resulted in a high level of variability in state- and county-level policy responses. This situation now allows for comparative analyses to inform effective policy making.

This cohort study provides one such comparative analysis, suggesting that government implementation of emergent measures—such as nursing home and prison visitation restrictions, school closures, mask mandates, and jail decarceration—are important for effective epidemic mitigation. Furthermore, its findings reflect that epidemic control depends not only on emergent responses but also on longer-term policy determinants of public health vulnerability. Specifically, our results suggest that the globally unparalleled system of mass incarceration in the US, which is known to incubate infectious diseases and to spread them to broader communities, puts the entire country at distinctive epidemiologic risk. This study is thus consistent with existing expert consensus^16^ that public investment in a national program of large-scale decarceration and reentry support is an essential policy priority for reducing racial inequality and improving US public health and safety, pandemic preparedness, and biosecurity.

## Notes

Supplement. **eAppendix 1.** Robustness Checks **eAppendix 2.** Limitations **eTable 1.** Estimated Relationships Between COVID-19 Daily Growth Rate and Log Daily Jail Population and Anticontagion Policies in Multivariate Regression Analysis With County Fixed Effects **eTable 2.** Estimated Relationships Between Growth Rate in COVID-19 Cases and Log Imputed Daily Jail Cycling and All Intervention Policies in Multivariate Regression Analysis With County Fixed Effects **eTable 3.** Estimated Relationships Between COVID-19 Daily Growth Rate and Log Daily Jail Population and Anticontagion Policies in Multivariate Regression Analysis With County Fixed Effects Using Data up to August 31, 2020 **eTable 4.** Estimated Relationships Between COVID-19 Daily Growth Rate and All the Anti-Contagion Policies in Multivariate Regression Analysis With County Fixed Effects **eFigure 1.** The Relationship Between Daily Jail Population and Growth in COVID-19 **eTable 5.** Estimated Relationships Between Growth Rate in COVID-19 Cases and Log Jail Population and All Intervention Policies in Multivariate Regression Analysis With County Fixed Effects and One-Week Lag Between Intervention and COVID-19 Growth Rate **eTable 6.** The Relationship Between COVID-19 Case Growth Rate and Jail Population and All Policy Variables When Accounting for the Effects of COVID-19 Daily Growth Rates Two Weeks Ago on Subsequent COVID-19 Daily Growth Rates **eFigure 2.** The Cumulative Plot of t-values Obtained From Regressions Dropping One State-Week (ie, One Week of One State) at a Time From the Panel Dataset **eFigure 3.** We Plot the Bivariate Correlation Between Log Jail Population and COVID-19 Growth With County Fixed Effects by Varying the Lag Periods Applied on the Log Jail Population Term (1, 2, 3 and 4 Weeks) **eFigure 4.** Association Between COVID-19 Growth Rate and Jail Population **eTable 7.** Comparison of Demographic Characteristics Between the Counties Included in Our Analytical Sample (In-Sample) and the Counties That Were Not Included Owing to the Lack of Jail Population Data (Out-Sample)
