# Supplementary material for: Association of Jail Decarceration and Anticontagion Policies With COVID-19 Case Growth Rates in US Counties
Source: JAMA Netw Open. 2021 Sep 2;4(9):e2123405. doi: 10.1001/jamanetworkopen.2021.23405 (PMC8414192; doi:10.1001/jamanetworkopen.2021.23405)
Supplement: Supplement. — eAppendix 1. Robustness Checks eAppendix 2. Limitations eTable 1. Estimated Relationships Between COVID-19 Daily Growth Rate and Log Daily Jail Population and Anticontagion Policies in Multivariate Regression Analysis With County Fixed Effects eTable 2. Estimated Relationships Between Growth Rate in COVID-19 Cases and Log Imputed Daily Jail Cycling and All Intervention Policies in Multivariate Regression Analysis With County Fixed Effects eTable 3. Estimated Relationships Between COVID-19 Daily Growth Rate and Log Daily Jail Population and Anticontagion Policies in Multivariate Regression Analysis With County Fixed Effects Using Data up to August 31, 2020 eTable 4. Estimated Relationships Between COVID-19 Daily Growth Rate and All the Anti-Contagion Policies in Multivariate Regression Analysis With County Fixed Effects eFigure 1. The Relationship Between Daily Jail Population and Growth in COVID-19 eTable 5. Estimated Relationships Between Growth Rate in COVID-19 Cases and Log Jail Population and All Intervention Policies in Multivariate Regression Analysis With County Fixed Effects and One-Week Lag Between Intervention and COVID-19 Growth Rate eTable 6. The Relationship Between COVID-19 Case Growth Rate and Jail Population and All Policy Variables When Accounting for the Effects of COVID-19 Daily Growth Rates Two Weeks Ago on Subsequent COVID-19 Daily Growth Rates eFigure 2. The Cumulative Plot of t-values Obtained From Regressions Dropping One State-Week (ie, One Week of One State) at a Time From the Panel Dataset eFigure 3. We Plot the Bivariate Correlation Between Log Jail Population and COVID-19 Growth With County Fixed Effects by Varying the Lag Periods Applied on the Log Jail Population Term (1, 2, 3 and 4 Weeks) eFigure 4. Association Between COVID-19 Growth Rate and Jail Population eTable 7. Comparison of Demographic Characteristics Between the Counties Included in Our Analytical Sample (In-Sample) and the Counties That Were Not Included Owing to the Lack o [file jamanetwopen-e2123405-s001.pdf]

Supplementary Online Content

Reinhart E, Chen DL. Association of jail decarceration and anticontagion policies with COVID-19 case growth rates in US counties. *JAMA Netw Open*. 2021;4(9):e2123405. doi:10.1001/jamanetworkopen.2021.23405

eAppendix 1. Robustness Checks

eAppendix 2. Limitations

eTable 1. Estimated Relationships Between COVID-19 Daily Growth Rate and Log Daily Jail Population and Anticontagion Policies in Multivariate Regression Analysis With County Fixed Effects

eTable 2. Estimated Relationships Between Growth Rate in COVID-19 Cases and Log Imputed Daily Jail Cycling and All Intervention Policies in Multivariate Regression Analysis With County Fixed Effects

eTable 3. Estimated Relationships Between COVID-19 Daily Growth Rate and Log Daily Jail Population and Anticontagion Policies in Multivariate Regression Analysis With County Fixed Effects Using Data up to August 31, 2020

eTable 4. Estimated Relationships Between COVID-19 Daily Growth Rate and All the Anti-Contagion Policies in Multivariate Regression Analysis With County Fixed Effects

eFigure 1. The Relationship Between Daily Jail Population and Growth in COVID-19

eTable 5. Estimated Relationships Between Growth Rate in COVID-19 Cases and Log Jail Population and All Intervention Policies in Multivariate Regression Analysis With County Fixed Effects and One-Week Lag Between Intervention and COVID-19 Growth Rate

eTable 6. The Relationship Between COVID-19 Case Growth Rate and Jail Population and All Policy Variables When Accounting for the Effects of COVID-19 Daily Growth Rates Two Weeks Ago on Subsequent COVID-19 Daily Growth Rates

eFigure 2. The Cumulative Plot of t-values obtained From Regressions Dropping One State-Week (ie, One Week of One State) at a Time From the Panel Dataset

eFigure 3. We Plot the Bivariate Correlation Between Log Jail Population and COVID-19 Growth With County Fixed Effects by Varying the Lag Periods Applied on the Log Jail Population Term (1, 2, 3 and 4 Weeks)

eFigure 4. Association Between COVID-19 Growth Rate and Jail Population

eTable 7. Comparison of Demographic Characteristics Between the Counties Included in Our Analytical Sample (In-Sample) and the Counties That Were Not Included Owing to the Lack of Jail Population Data (Out-Sample)

This supplementary material has been provided by the authors to give readers additional information about their work.

© 2021 Reinhart E et al. *JAMA Network Open.*

eAppendix 1. Robustness Checks

Some policies in our dataset do not feature dates of change after August 31, introducing concern for possible measurement error. We therefore conduct an additional analysis that restricts to an end-date of August 31 in order to investigate whether, when controlling for anti-contagion policies without this possible measurement error, daily jail population remains correlated with COVID-19 daily growth rates both quadratically and linearly. The results of this analysis, which can be found in eTable 3, are consistent with our broader analysis.

As an additional analysis of anti-contagion policies and as a robustness check to evaluate possible selection bias in our analysis sample, we drop the jail variables in order to evaluate anti-contagion policies for all counties without restricting to those for which we have jail population data. This increases our number of observations from 319,084 (when the sample is restricted to only those counties for which we have jail data) to 725,407 (when unrestricted by jail data availability). The results of this analysis (see eTable 4) are broadly consistent with the results reported below for our analysis restricted to counties for which we have jail data; the estimates in Column 2 are roughly a factor of 0.8 of those in Column 1, with the exception of two policies in which the difference is about the same magnitude but in the other direction. This mitigates concerns regarding selection bias.

An additional way to assuage possible concerns for outliers in our sample is to drop one state-week at a time, as presented in eFigure 2. This figure, in combination with Figure 1, provide a robustness check for possible outliers that might account for our results.

eTable 5 repeats the regressions from Table 2 while using a one-week lag between intervention and COVID-19 growth rate rather than a two-week lag. The patterns observed with this one-week lag are larger by a factor of 1.4 for jail population; the remaining coefficients remain numerically very similar to the patterns reported in Table 2. eFigure 3 presents binned scatter plots for each of four lags between jail population and COVID-19 growth rate.

© 2021 Reinhart E et al. *JAMA Network Open.*

As seen in eTable 6, the association between daily jail population and daily COVID-19 growth rate two weeks later persists even when controlling for a two-week lag of COVID-19 growth—that is, when controlling for the association of earlier COVID-19 growth rates on subsequent COVID-19 growth rates. Jail cycling is expected to function as multiplier of incoming COVID-19 cases from the community that then disseminate back to community contexts in a positive feedback loop that compounds community COVID-19 growth rates.[18] Consistent with this expectation, controlling for this lagged COVID-19 growth diminishes the magnitude of the observed coefficients for the effects of jail population. The quadratic relationship nonetheless remains statistically significant ($p < 0.001$) with this additional control.

In eFigure 2, to assuage concerns of spatial or temporal outliers driving our main findings, we drop one state-week (i.e., one week of one state) at a time and present the coefficient estimates corresponding to the linear model in Table 2, column 2. All values were above conventional significance levels.

Figures S4A-B also display the relationship separately by time period and intensity of SARS-CoV-2 outbreak in a county. The patterns we observe are also much larger when caseloads are higher. Accordingly, the patterns we observe are much larger from April to August 2020, prior temporary decline in US COVID-19 cases in the autumn months before their rapid increases beginning in November. Notably the patterns are large prior to the adoption of any anti-contagion policy in a given county (i.e., when jail cycling policies cannot be associated with other anti-contagion policy measures).

eTable 7 presents a comparison of demographic characteristics between the counties included in our analytical sample (in-sample) and the counties that were not included owing to the lack of jail population data (out-sample). We performed a two-sample t-test between the two sets of counties on selected demographic variables. The analysis sample is slightly biased towards minority, poorer, and younger populations.

© 2021 Reinhart E et al. *JAMA Network Open.*

eAppendix 2. Limitations

We are limited by the non-universal coverage of data on county-level policy interventions. Our sources allow us to observe 1,144 counties—approximately 72% of all counties in our analysis sample—with at least one policy variable in addition to state-wide CUSP variables. More specifically, we use county-specific, non-CUSP-reported data on stay-at-home policies for 554 counties; restaurant closures for 332 counties; non-essential business closures for 591 counties; and school closures for 481 counties. Our data do not include intra-state county-level variation for other variables such as mask mandates, gym closures, or prohibitions on nursing home visitation.

© 2021 Reinhart E et al. *JAMA Network Open.*

eTable 1. Estimated relationships between COVID-19 daily growth rate and log daily jail population and anti-contagion policies in multivariate regression analysis with county fixed effects.

**Independent variable**	**Coefficients for growth rate of COVID-19 cases (Cubic) [95 % CI]**	**Coefficients for growth rate of COVID-19 cases (Quadratic) [95 % CI]**	**Coefficients for growth rate of COVID-19 cases (Linear) [95 % CI]**
Log daily jail population 2 weeks ago	0.027*** [0.013,0.041]	-0.020** [-0.031,-0.008]	0.008*** [0.003,0.012]
Log daily jail population (squared) 2 weeks ago	- 0.012*** [-0.018,-0.007]	0.004*** [0.002,0.006]	
Log daily jail population (cubed) 2 weeks ago	0.002*** [0.001,0.002]		
Nursing home visitation ban policy in effect 2 weeks ago	-0.073*** [-0.087,-0.058]	-0.073*** [-0.088,-0.058]	-0.074*** [-0.089,-0.058]
Schools closure policy in effect 2 weeks ago	-0.042*** [-0.065,-0.020]	-0.043*** [-0.066,-0.020]	-0.046*** [-0.069,-0.022]
Mandatory mask policy (state-wide) in effect 2 weeks ago	-0.024*** [-0.032,-0.016]	-0.025*** [-0.033,-0.017]	-0.025*** [-0.033,-0.017]
Prison visitation ban policy in effect 2 weeks ago	-0.012* [-0.022,-0.003]	-0.012* [-0.022,-0.003]	-0.013** [-0.023,-0.003]
Stay-at-home policy in effect 2 weeks ago	-0.007+ [-0.016,0.001]	-0.008+ [-0.016,0.001]	-0.008+ [-0.017,0.001]
Other non-essential businesses closure policy in effect 2 weeks ago	-0.005 [-0.018,0.007]	-0.005 [-0.018,0.007]	-0.006 [-0.018,0.007]
Gyms closure policy in effect 2 weeks ago	-0.005 [-0.013,0.003]	-0.005 [-0.013,0.003]	-0.006 [-0.014,0.002]
Bars closure policy in effect 2 weeks ago	-0.003 [-0.011,0.005]	-0.003 [-0.011,0.005]	-0.003 [-0.010,0.005]
Movie theatres closure policy in effect 2 weeks ago	-0.001 [-0.009,0.007]	-0.001 [-0.009,0.007]	-0.002 [-0.010,0.007]
Restaurants closure policy in effect 2 weeks ago	0.022*** [0.010,0.034]	0.022*** [0.011,0.033]	0.023*** [0.012,0.035]

Note: The following symbols indicate the significance level (p-value) of the coefficients:
***** p<0.001 ** p<0.01 *p<0.05 +p<0.1**

The table compares, as a robustness check, estimates from regressions using a cubic polynomial term on jail population variable with that of the quadratic and linear regressions which we tabulated in Table T2. We can see that the COVID-19 case growth rate, upon adding a cubic polynomial term also finds a significant positive association with jail population.

© 2021 Reinhart E et al. *JAMA Network Open.*

eTable 2. Estimated relationships between growth rate in COVID-19 cases and log imputed daily jail cycling and all intervention policies in multivariate regression analysis with county fixed effects.

**Independent variable**	**Coefficients for growth rate of COVID-19 cases (Quadratic) [95 % CI]**	**Coefficients for growth rate of COVID-19 cases (Linear) [95 % CI]**
Log jail cycling 2 weeks ago	-0.030*** [-0.046,-0.015]	0.017*** [0.009,0.024]
Log jail cycling (squared) 2 weeks ago	0.014*** [0.009,0.020]	
Nursing home visitation ban policy in effect 2 weeks ago	-0.073*** [-0.088,-0.058]	-0.073*** [-0.089,-0.058]
Schools closure policy in effect 2 weeks ago	-0.043*** [-0.065,-0.020]	-0.045*** [-0.068,-0.022]
Mandatory mask policy (state-wide) in effect 2 weeks ago	-0.024*** [-0.032,-0.017]	-0.025*** [-0.033,-0.017]
Prison visitation ban policy in effect 2 weeks ago	-0.012* [-0.022,-0.003]	-0.013** [-0.023,-0.003]
Stay-at-home policy in effect 2 weeks ago	-0.008+ [-0.016,0.001]	-0.008+ [-0.016,0.001]
Other non-essential businesses closure policy in effect 2 weeks ago	-0.005 [-0.018,0.007]	-0.006 [-0.018,0.007]
Gyms closure policy in effect 2 weeks ago	-0.005 [-0.013,0.003]	-0.006 [-0.014,0.002]
Bars closure policy in effect 2 weeks ago	-0.003 [-0.011,0.005]	-0.003 [-0.010,0.005]
Movie Theaters closure policy in effect 2 weeks ago	-0.001 [-0.009,0.007]	-0.001 [-0.010,0.007]
Restaurants closure policy in effect 2 weeks ago	0.022*** [0.010,0.034]	0.023*** [0.012,0.034]

Note: The following symbols indicate the significance level (p-value) of the coefficients:
***** p<0.001 ** p<0.01 *p<0.05 +p<0.1**

© 2021 Reinhart E et al. *JAMA Network Open.*

eTable 3. Estimated relationships between COVID-19 daily growth rate and log daily jail population and anti-contagion policies in multivariate regression analysis with county fixed effects using data up to August 31, 2020.

**Independent variable**	**Coefficients for growth rate of COVID-19 cases (Quadratic) [95 % CI]**	**Coefficients for growth rate of COVID-19 cases (Linear) [95 % CI]**
Log Daily Jail Population 2 weeks ago	-0.040*** [-0.057,-0.024]	0.020*** [0.012,0.028]
Log Daily Jail Population (squared) 2 weeks ago	0.009*** [0.006,0.012]	
Nursing home visitation ban policy in effect 2 weeks ago	-0.064*** [-0.077,-0.052]	-0.066*** [-0.079,-0.052]
Schools closure policy in effect 2 weeks ago	-0.042*** [-0.061,-0.022]	-0.047*** [-0.067,-0.026]
Mandatory mask policy (state-wide) in effect 2 weeks ago	-0.023*** [-0.031,-0.015]	-0.024*** [-0.033,-0.015]
Prison visitation ban policy in effect 2 weeks ago	-0.015** [-0.026,-0.005]	-0.018** [-0.029,-0.007]
Stay-at-home policy in effect 2 weeks ago	-0.009+ [-0.017,-0.001]	-0.010* [-0.018,-0.002]
Other non-essential businesses closure policy in effect 2 weeks ago	-0.006 [-0.018,0.005]	-0.006 [-0.018,0.006]
Movie theatres closure policy in effect 2 weeks ago	-0.004 [-0.011,0.002]	-0.005 [-0.012,0.002]
Bars closure policy in effect 2 weeks ago	-0.002 [-0.007,0.004]	-0.001 [-0.007,0.004]
Gyms closure policy in effect 2 weeks ago	-0.003 [-0.010,0.004]	-0.004 [-0.012,0.003]
Restaurants closure policy in effect 2 weeks ago	0.021*** [0.011,0.032]	0.023*** [0.013,0.034]

Note: The following symbols indicate the significance level (p-value) of the coefficients:
***** p<0.001 ** p<0.01 *p<0.05 +p<0.1**

© 2021 Reinhart E et al. *JAMA Network Open.*

This table shows that when restricting the data to August 31 (after which some of the policy variables are not updated in our data sources as inactive if they have been lifted during this period), even when controlling—without measurement error in data after August 31—for anti-contagion policies, daily jail population correlates with COVID-19 daily growth rates both quadratically in column 1 and linearly in column 2. The association between jail population and COVID-19 daily growth rates appears to grow quadratically.

© 2021 Reinhart E et al. *JAMA Network Open.*

eTable 4. Estimated relationships between COVID-19 daily growth rate and all the anti-contagion policies in multivariate regression analysis with county fixed effects.

**Independent variable**	**Coefficients for growth rate of COVID-19 cases (Restricted by jail data availability) [95 % CI] [no of obs = 319084]**	**Coefficients for growth rate of COVID-19 cases [95 % CI] [no of obs = 725407]**
Nursing home visitation ban policy in effect 2 weeks ago	-0.072*** [-0.088,-0.057]	-0.064*** [-0.094,-0.035]
Schools closure policy in effect 2 weeks ago	-0.047*** [-0.070,-0.024]	-0.036** [-0.059,-0.013]
Mandatory mask policy (state-wide) in effect 2 weeks ago	-0.025*** [-0.033,-0.017]	-0.024*** [-0.031,-0.017]
Prison visitation ban policy in effect 2 weeks ago	-0.014** [-0.024,-0.004]	-0.013* [-0.025,-0.001]
Stay-at-home policy in effect 2 weeks ago	-0.008+ [-0.017,0.000]	-0.010* [-0.019,-0.001]
Other non-essential businesses closure policy in effect 2 weeks ago	-0.006 [-0.018,0.007]	0.000 [-0.010,0.009]
Gyms closure policy in effect 2 weeks ago	-0.006 [-0.014,0.002]	-0.006* [-0.012,0.000]
Bars closure policy in effect 2 weeks ago	-0.003 [-0.010,0.005]	0.000 [-0.007,0.006]
Movie theatres closure policy in effect 2 weeks ago	-0.002 [-0.011,0.006]	-0.005 [-0.011,0.001]
Restaurants closure policy in effect 2 weeks ago	0.024*** [0.013,0.035]	0.022*** [0.012,0.032]

Note: The following symbols indicate the significance level (p-value) of the coefficients:
***** p<0.001 ** p<0.01 *p<0.05 +p<0.1**

eTable 4 shows that the association between policies and COVID-19 growth remain similar when, in Column 1, we remove daily jail population as a control but keep the sample of counties for which we have data on jail population, and in Column 2, when include all US counties without restricting to

availability of data on jail populations. (For further demographic detail on the counties in our sample, see eTable 6.)

eFigure 1A. The Relationship Between Daily Jail Population and Growth in COVID-19 is substantially larger in counties with above median Black population compared to counties with below median Black population—we plot the bivariate correlation between log jail population two weeks ago and COVID-19 growth with county fixed effects for the two samples.

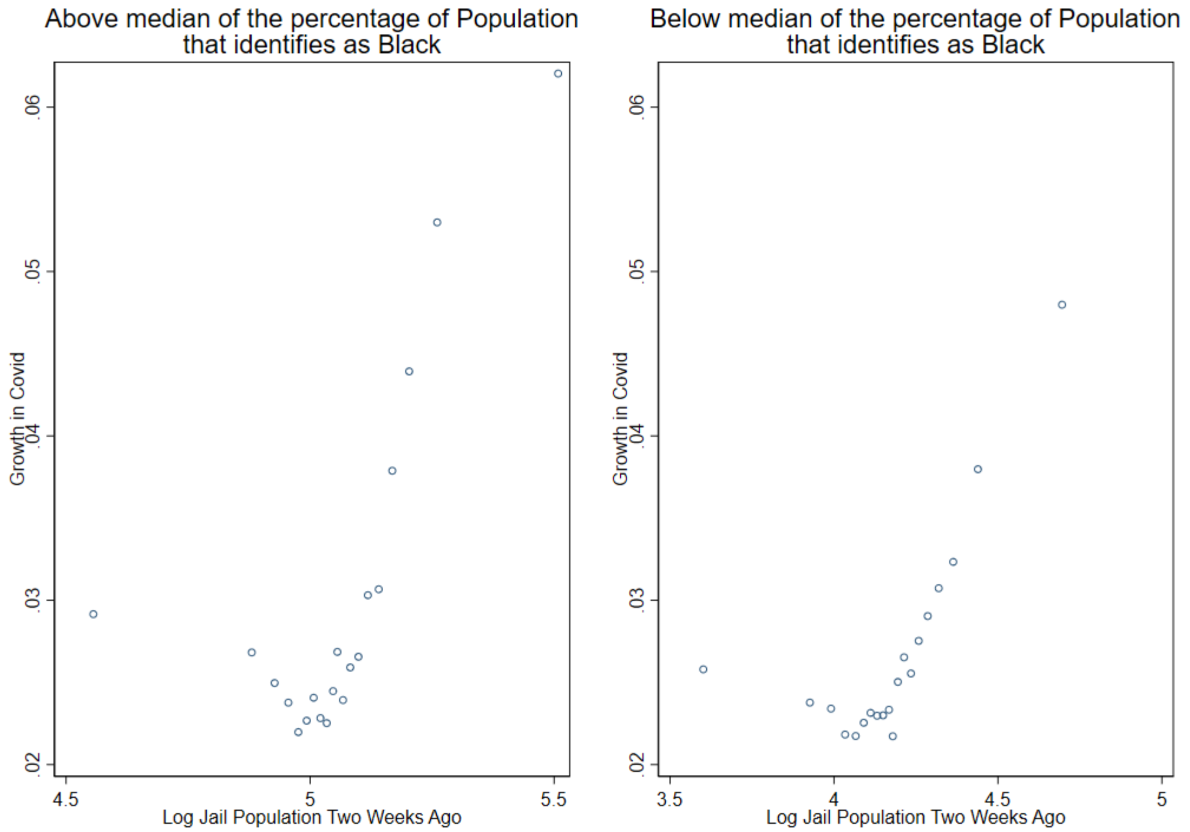

© 2021 Reinhart E et al. *JAMA Network Open.*

eFigure 1B. The relationship between daily jail population and growth in COVID-19 is substantially larger in counties with above median of county-median household income compared to counties with below median of county median household income—we plot the bivariate correlation between log jail population two weeks ago and COVID-19 growth with county fixed effects for the two samples.

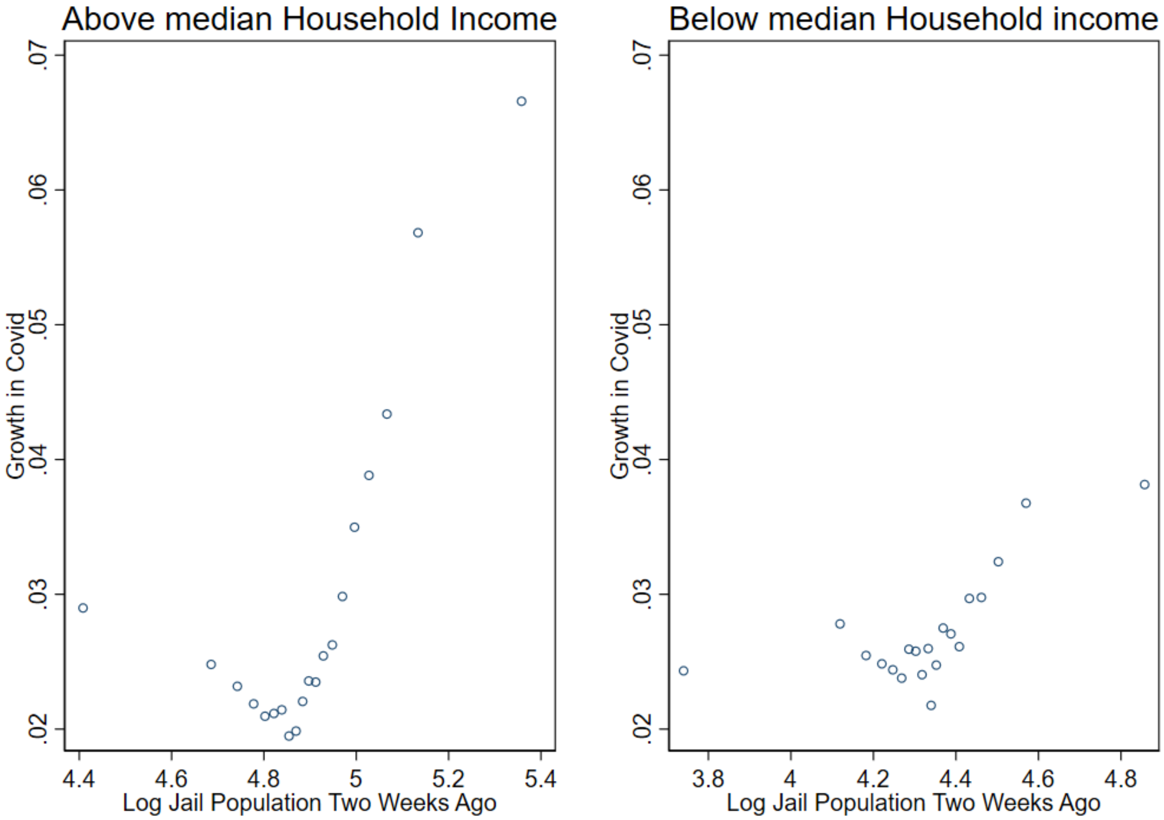

© 2021 Reinhart E et al. *JAMA Network Open.*

eFigure 1C. The relationship between jail population and growth in COVID-19 is substantially larger in the top 50 counties by population – we plot the bivariate correlation between log jail population two weeks ago and COVID-19 growth with county fixed effects for the two samples.

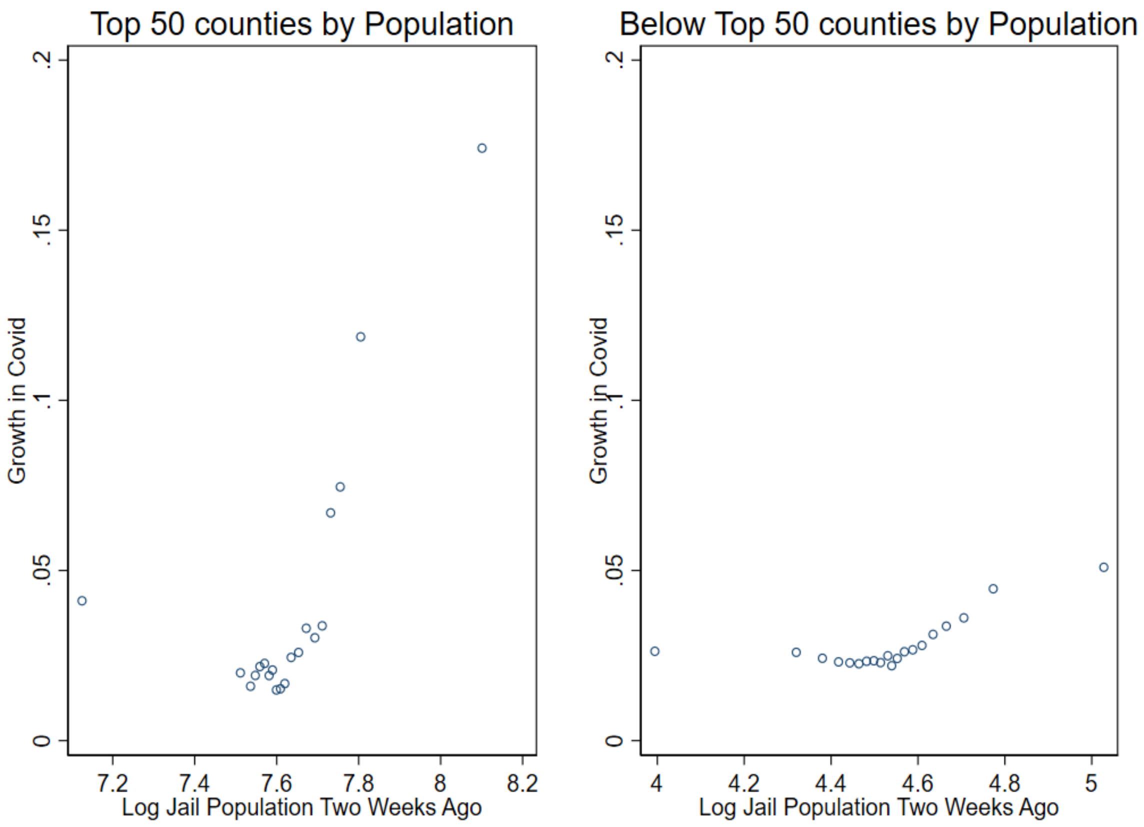

© 2021 Reinhart E et al. *JAMA Network Open.*

eTable 5. Estimated relationships between growth rate in COVID-19 cases and log jail population and all intervention policies in multivariate regression analysis with county fixed effects and one-week lag between intervention and COVID-19 growth rate.

**Independent variable**	**Coefficients for growth rate of COVID-19 cases (Quadratic) [95 % CI]**	**Coefficients for growth rate of COVID-19 cases (Linear) [95 % CI]**
Log Daily Jail population 1 week ago	-0.022** [-0.037,-0.007]	0.011*** [0.005,0.017]
Log Daily Jail population (squared) 1 week ago	0.005*** [0.002,0.009]	
Nursing home visitation ban policy in effect 1 week ago	-0.093*** [-0.109,-0.077]	-0.093*** [-0.110,-0.077]
Schools closure policy in effect 1 week ago	-0.026* [-0.052,-0.001]	-0.029* [-0.056,-0.003]
Mandatory mask policy (state-wide) in effect 1 week ago	-0.025*** [-0.034,-0.015]	-0.025*** [-0.034,-0.015]
Prison visitation ban policy in effect 1 week ago	-0.003 [-0.012,0.006]	-0.004 [-0.013,0.005]
Stay-at-home policy in effect 1 week ago	-0.011+ [-0.023,0.001]	-0.011+ [-0.023,0.001]
Other non-essential businesses closure policy in effect 1 week ago	0.000 [-0.015,0.015]	0.000 [-0.015,0.015]
Gyms closure policy in effect 1 week ago	- 0.003 [-0.012,0.006]	-0.004 [-0.013,0.005]
Bars closure policy in effect 1 week ago	0.000 [-0.009,0.009]	0.000 [-0.009,0.008]
Movie theatres closure policy in effect 1 week ago	-0.006 [-0.016,0.004]	-0.007 [-0.017,0.004]
Restaurants closure policy in effect 1 week ago	0.030*** [0.017,0.042]	0.031*** [0.019,0.043]

Note: The following symbols indicate the significance level (p-value) of the coefficients:
***** p<0.001** **** p<0.01** ***p<0.05** **+p<0.1**

© 2021 Reinhart E et al. *JAMA Network Open.*

eTable 6. The relationship between COVID-19 case growth rate and jail population and all policy variables when accounting for the effects of COVID-19 daily growth rates two weeks ago on subsequent COVID-19 daily growth rates.

**Independent variable**	**Coefficients for growth rate of COVID-19 cases (Quadratic) [95 % CI]**	**Coefficients for growth rate of COVID-19 cases (Linear) [95 % CI]**
Log Daily Jail Population 2 weeks ago	-0.006 [-0.015,0.002]	0.004** [0.001,0.007]
Log Daily Jail Population (squared) 2 weeks ago	0.002* [0.000,0.003]	
Nursing home visitation ban policy in effect 2 weeks ago	-0.064*** [-0.083,-0.046]	-0.064*** [-0.083,-0.045]
Schools closure policy in effect 2 weeks ago	-0.025+ [-0.050,0.000]	-0.026* [-0.051,-0.001]
Mandatory mask policy (state-wide) in effect 2 weeks ago	-0.017*** [-0.022,-0.013]	-0.017*** [-0.022,-0.013]
Prison visitation ban policy in effect 2 weeks ago	-0.007* [-0.013,-0.001]	-0.007* [-0.013,-0.002]
Stay-at-home policy in effect 2 weeks ago	-0.002 [-0.009,0.005]	-0.002 [-0.009,0.005]
Other non-essential businesses closure policy in effect 2 weeks ago	0.001 [-0.007,0.008]	0.001 [-0.007,0.008]
Gyms closure policy in effect 2 weeks ago	-0.004 [-0.008,-0.001]	-0.004+ [-0.009,-0.001]
Bars closure policy in effect 2 weeks ago	-0.004 [-0.010,0.002]	-0.004 [-0.010,0.002]
Movie theatres closure policy in effect 2 weeks ago	0.001 [-0.004,0.005]	0.001 [-0.004,0.005]
Restaurants closure policy in effect 2 weeks ago	0.009* [0.002,0.016]	0.009* [0.002,0.016]
Growth in COVID-19 cases 2 weeks ago	0.077*** [0.062,0.091]	0.077*** [0.062,0.093]

© 2021 Reinhart E et al. *JAMA Network Open.*

Note: The following symbols indicate the significance level (p-value) of the coefficients:

***** p<0.001 ** p<0.01 *p<0.05 +p<0.1**

© 2021 Reinhart E et al. *JAMA Network Open.*

eFigure 2. The cumulative plot of t-values obtained from regressions dropping one state-week (i.e., one week of one state) at a time from the panel dataset. The linear regression model uses the variables corresponding to column 2 of Table 2 in the main text.

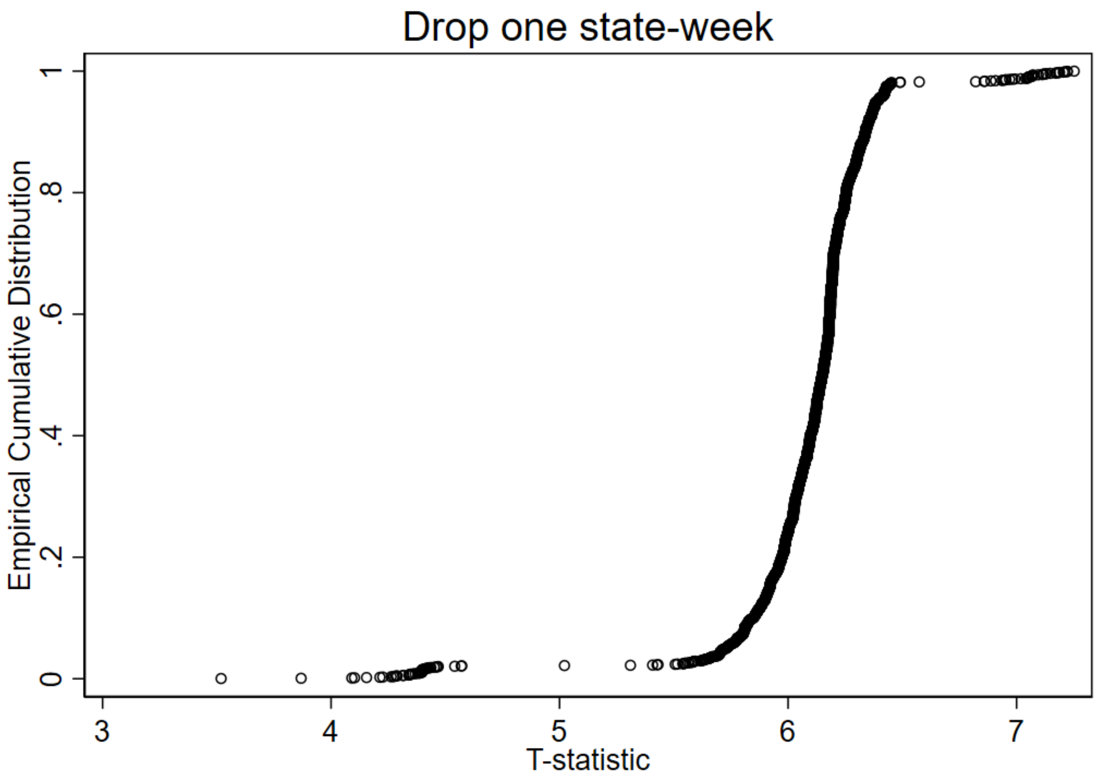

© 2021 Reinhart E et al. *JAMA Network Open.*

eFigure 3. We plot the bivariate correlation between log jail population and COVID-19 growth with county fixed effects by varying the lag periods applied on the log jail population term (1, 2, 3 and 4 weeks). We can see that the COVID growth rate has a higher association with jail population 2 weeks ago than a week ago. This is in line with the estimates observed in Table 2 and eTable 5.

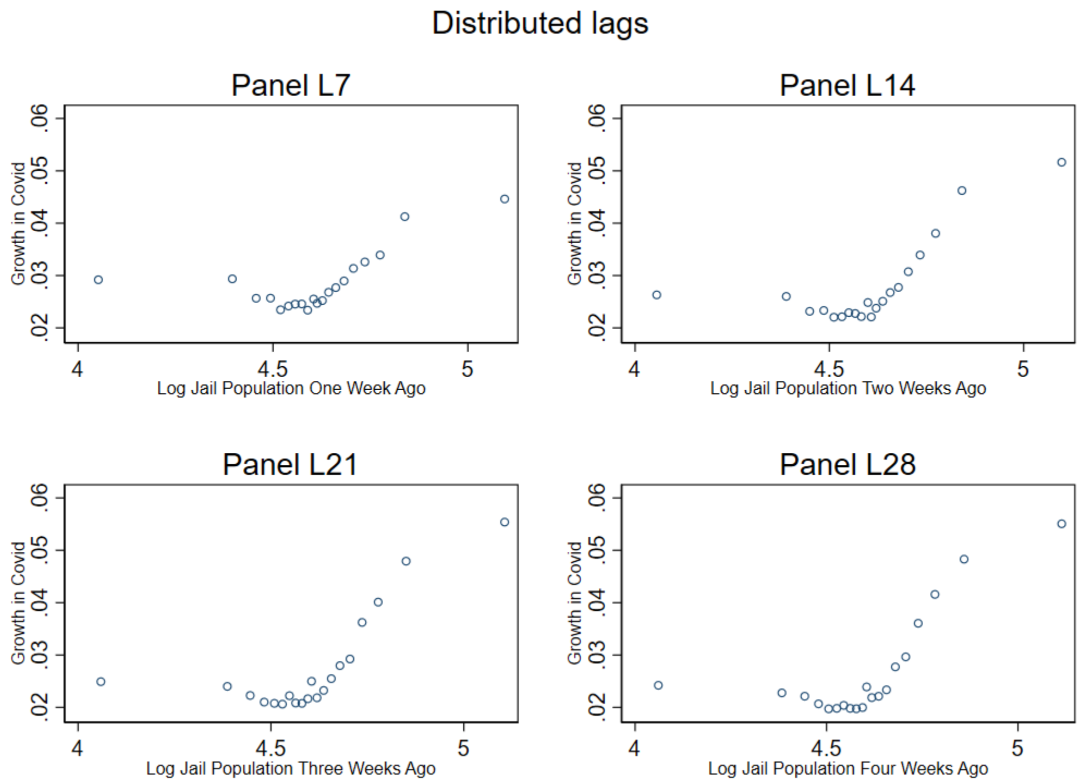

© 2021 Reinhart E et al. *JAMA Network Open.*

eFigure 4A. Shows that the COVID-19 growth rate's association with jail population is higher when the cumulative cases (i.e., epidemic size) are lower. This reflects our definition of daily growth rate: new cases on a given day / cumulative cases on the previous day**.** We plot the bivariate correlation between log jail population 2 weeks ago and COVID-19 growth with county fixed effects.

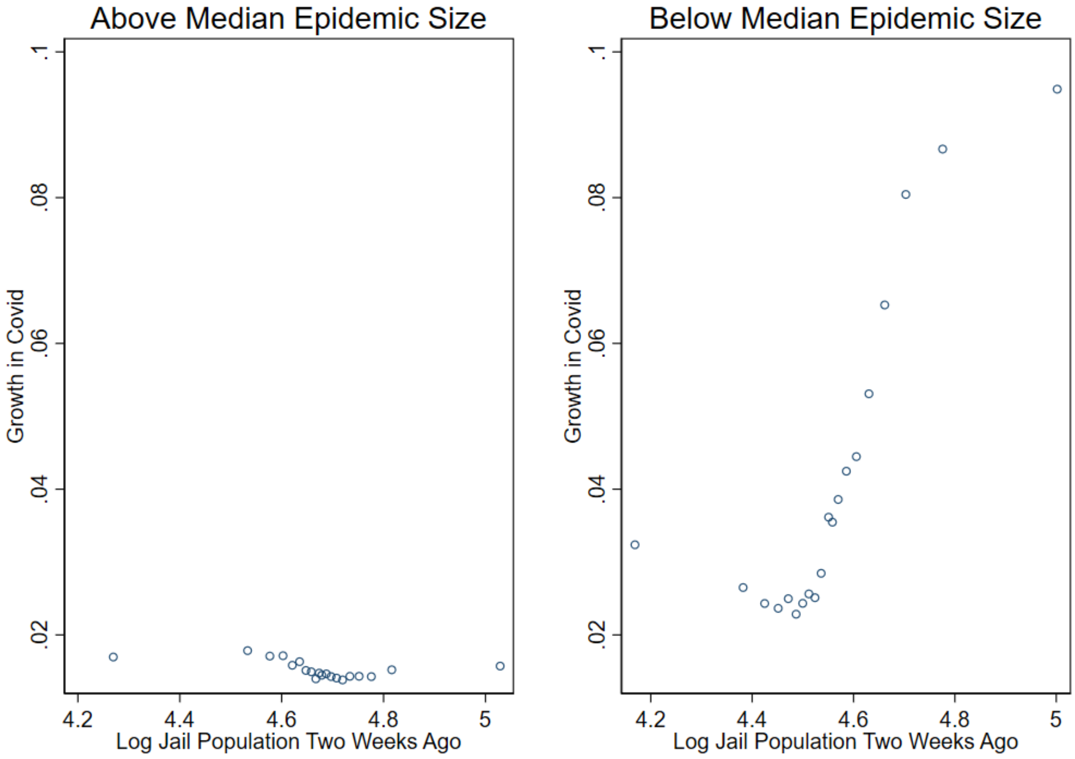

© 2021 Reinhart E et al. *JAMA Network Open.*

eFigure 4B. Shows the relationship between COVID-19 growth rate and jail population across three different epidemic time periods: March, April to August, September to November 15. Noticeably, the growth rate associated with jail population persists except in the first full month of the U.S. epidemic**.** We plot the bivariate correlation between log jail population 2 weeks ago and COVID-19 growth with county fixed effects.

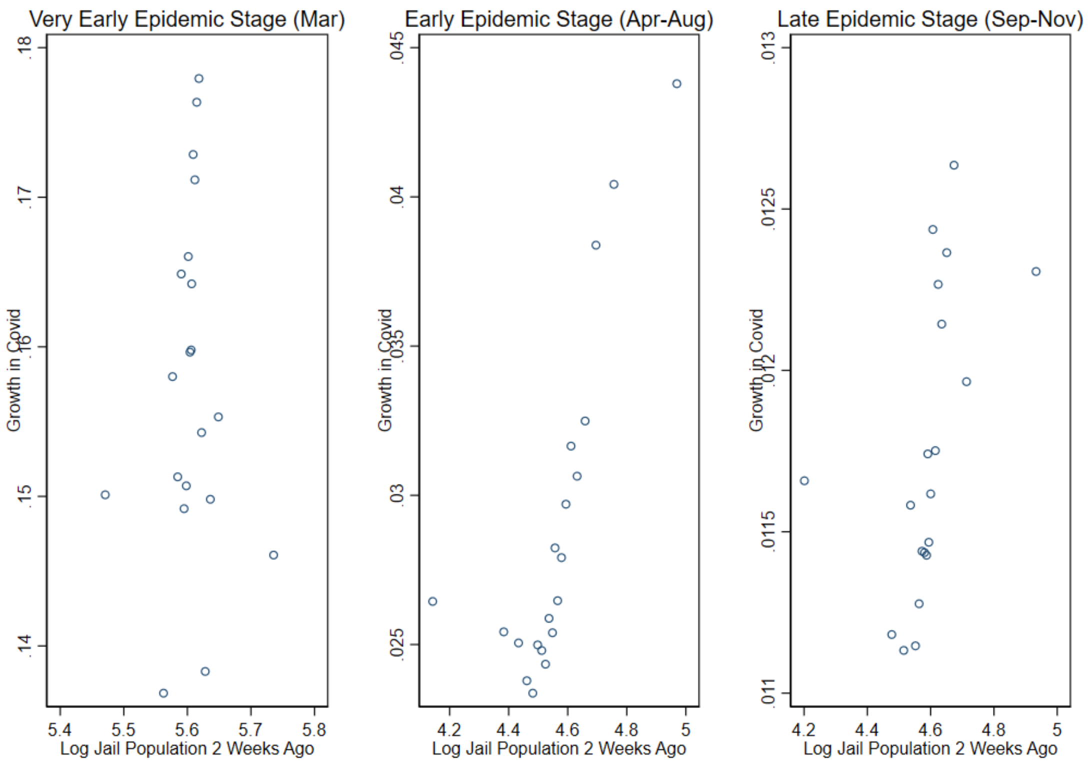

© 2021 Reinhart E et al. *JAMA Network Open.*

eTable 7. Comparison of demographic characteristics between the counties included in our analytical sample (in-sample) and the counties that were not included owing to the lack of jail population data (out-sample). We performed a two-sample t-test between the two sets of counties on selected demographic variables. The analysis sample leans somewhat more heavily to minority, poorer, and younger populations.

Demographic Variable	All Sample Mean	Out-sample Mean	In-sample Mean	Difference of Means	p-value	Total count	Out-sample count	In-sample count
Black residents per capita	0.093	0.081	0.105	-0.024***	0.000	3140	1535	1605
Proportion of total population living under poverty	0.152	0.148	0.155	-0.007**	0.002	3140	1535	1605
Population density	283.148	259.115	307.317	-48.202	0.427	3219	1614	1605
Hispanic residents per capita	0.096	0.068	0.123	-0.055***	0.000	3140	1535	1605
Median household income	52800.804	52527.905	53061.801	-533.896	0.281	3140	1535	1605
Proportion of total population aged 65 and above	0.193	0.201	0.185	0.016***	0.000	3141	1536	1605

Note: The race and ethnicity classifications used here are adopted from the US Census and American Community Survey.

© 2021 Reinhart E et al. *JAMA Network Open.*
